# Morphology, Histology, and Histochemistry of the Digestive Tract of the Marbled Flounder *Pseudopleuronectes yokohamae*

**DOI:** 10.3390/ani13050936

**Published:** 2023-03-05

**Authors:** Jeong-Hyeon Cho, Jin Woo Park, Yong-Woon Ryu, Kang-Woong Kim, Sang-Woo Hur

**Affiliations:** 1 Jeju Fisheries Research Institute, National Institute of Fisheries Science, Jeju 63610, Republic of Korea; 2 Aquafeed Research Center, National Institute of Fisheries Science, Pohang 37517, Republic of Korea

**Keywords:** marbled flounder, digestive tract, digestive physiology, immunohistochemistry, endocrine cells

## Abstract

**Simple Summary:**

The marbled flounder (*Pseudopleuronectes yokohamae*) is a fish species with high commercial value in Korea and has been gaining attention as a new aquaculture target. To clarify the digestive physiology and feeding habits of the marbled flounder, this study conducted morphological, histological, and histochemical analyses of its digestive tract. In conclusion, the feeding habits of marbled flounder are close to those of carnivorous fish. In addition, we found that the marbled flounder has a stomach with a structure suitable for eating small amounts of hard food, and that most of its digestion takes place in the anterior intestine portion. Our study presents the histological and histochemical characteristics of the digestive tract of the marbled flounder, providing basic knowledge for both physiological and nutritional studies.

**Abstract:**

This study investigated the morphological, histological, and histochemical characteristics of the digestive tract of the marbled flounder (*Pseudopleuronectes yokohamae*). The relative length of the gut of the marbled flounder digestive tract was 1.54 ± 0.10 (*n* = 20), and it had a simple stomach and 6–9 pyloric caeca. The mucosal folds of the marbled flounder digestive tract exhibited a general branched morphology. The thickness and mucosal fold length of the intestinal muscularis externa showed similar aspects in all areas. The thickness of the intestinal muscularis externa was the thickest in the posterior intestine portion, and the length of mucosal folds was the longest in the anterior intestine portion. It was indicated that food digested by gastric acid in the stomach moves to the anterior portion (including pyloric caeca) and mid portion of the intestine, ensuring effective stimulation of cholecystokinin (CCK)-producing cells. In addition, the distribution pattern of CCK-producing cells in the intestine was very similar to that of mucus-secreting goblet cells. The CCK-producing cells and goblet cells in the marbled flounder were well-adapted to promote optimal control of the digestive process. Based on the morphological and histochemical studies, it was concluded that the marbled flounder displays a digestive tract comparable to that of fish species with carnivorous habits.

## 1. Introduction

The digestive tract of fish is anatomically composed of a long hollow tube, and as in other vertebrates, it is functionally in charge of important processes such as digestion, absorption, and transportation of nutrients necessary for body growth from food [1,2]. While the digestive tract of most fish is generally composed of a series of tubes including the mouth, pharynx, esophagus, stomach, intestine, rectum, and anus, the shape and structure vary depending on the species and feeding habits [3]. In particular, due to the diversity of feeding and phylogenetic characteristics of the digestive tract of teleosts, many studies related to the morphological and structural characteristics and ontogenetic development of the digestive tract have been reported [4,5,6,7,8]. The diversity of morphological and structural characteristics of fish digestive tracts indicates that each species has different dietary and digestive physiology characteristics [9,10]. Therefore, to understand the diet and digestive physiology of fish, histological studies on digestive tract morphology and internal structure must be performed; and since the understanding of the fish digestive tract is related to fish nutrition, such studies can also contribute to fish management and conservation [10].

The digestive system is the largest endocrine organ in vertebrates [2]. Hormones and a wide range of signaling molecules secreted by various types of endocrine cells can rapidly and reversibly change the properties of the digestive system and other organ systems [11]. Energy and nutrients are provided in a sequential process, where complex polymers are absorbed across the apical membrane of epithelial cells, transported to the systemic circulation, and hydrolyzed into smaller molecules [12,13,14]. Digestive system function is regulated by various digestive hormones and substances. Among them, cholecystokinin (CCK) and mucus-secreting goblet cells play an important physiological role in the intestine of vertebrates, including fish. CCK, a gastrointestinal hormone, is a neurotransmitter peptide abundant in the brain and gut [15]. CCK plays an important role in pancreatic enzyme secretion, gallbladder contraction, amino acid and sugar transport regulation, and intestinal peristalsis regulation [15]. In addition, mucus-secreting goblet cells in the fish digestive tract produce lubricant on the surface of the mucous membrane, protecting the mucous membrane from damage caused by physical or chemical substances and digestive enzymes [16]. In addition, mucus secreted from goblet cells of vertebrates, including fish, plays an important role in the digestion and absorption of substances [16].

The marbled flounder (*Pseudopleuronectes yokohamae*), found throughout Korea, southern Hokkaido in Japan, and the East China Sea, is a fish species with high commercial value in both Korea and Japan [17,18] and has been gaining attention as a new aquaculture target [19]. Previous studies determined the marbled flounder to be a benthic fish that feeds on macrozoobenthos, such as Pelecypoda, Amphipoda, and Polychaete [19]; however, there have been few histological and histochemical studies concerning the digestive tract of species of the *Pseudopleuronectes* genus, including the marbled flounder. Interest in the digestive physiology of cultured fish is based on the expectation that the application of histological and histochemical knowledge to the development of aquaculture and husbandry prevents digestive disorders and helps in understanding the regulatory aspects of fish nutrition [20]. Therefore, this study aims to provide basic knowledge for physiological and nutritional studies by describing the histological and histochemical characteristics of the marbled flounder digestive tract. 

## 2. Materials and Methods

Sample collection was carried out at the Aquafeed Research Center (Pohang, Korea) of the National Institute of Fisheries Science (NIFS) of Korea in accordance with the Guidelines for Experimental Animals of NIFS Institutional Animal Care Use Committee (2019-NIFS-IACUC-13).

### 2.1. Specimens

Twenty marbled flounder juveniles, with average body length and body weight of 14.6 ± 0.5 cm and 56.6 ± 5.1 g, respectively, were obtained from the Aquafeed Research Center, Pohang, Korea. The first broodstock generation had been hatched in the Fisheries Resources Institute, Gyeongsangbuk-do, located near our research facility (ca. 45 km). Fish were kept in natural photoperiod and water temperature conditions. The collected specimens were anaesthetized with an overdose of 2-phenoxyethanol (150 ppm; Sigma-Aldrich, St. Louis, MO, USA) and their body lengths were measured. The body cavity was cut open through a mid-ventral incision and the digestive system, from the esophagus to the anus, along with the accessory organs, was removed through dissection from the body cavity. The length of the digestive tract was measured, and relative length of the gut (RLG) was calculated using the equation RLG = DL/SL, where DL is the digestive tract length (cm) and SL is the standard length (cm) (Table 1).

### 2.2. Histochemistry

**Figure 1:**
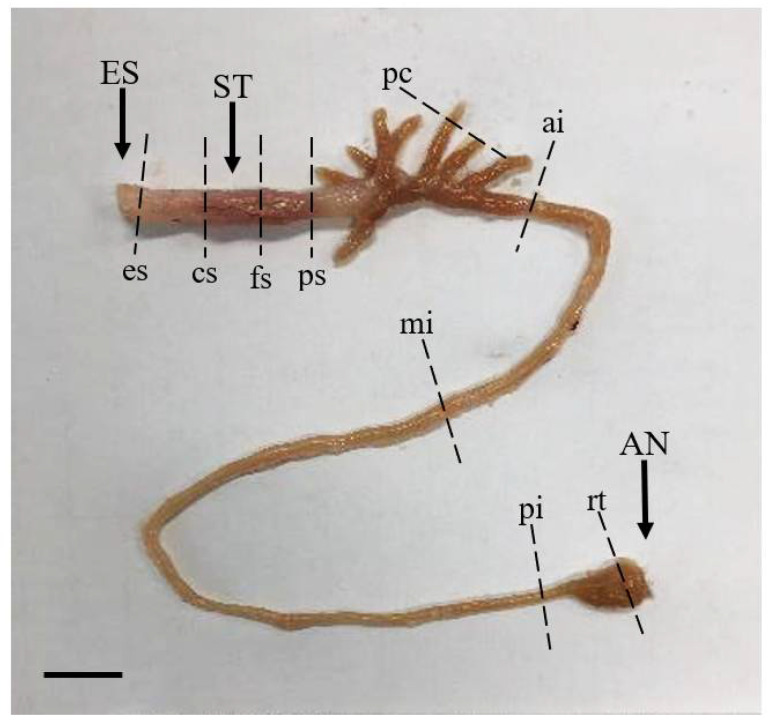
Anatomical features of the marbled flounder *Pseudopleuronectes yokohamae* digestive tract divided into nine segments. Abbreviations: ES, esophagus; ST, stomach; AN, anus; es, esophagus portion; cs, cardiac stomach portion; fs, fundic stomach portion; ps, pyloric stomach portion; pc, pyloric caeca portion; ai, anterior intestine portion; mi, mid intestine portion; pi, posterior intestine portion; rt, rectum portion. Scale bar: 10 mm.

Samples from nine different regions of the digestive tract (esophagus, cardiac stomach portion, fundic stomach portion, pyloric stomach portion, pyloric caeca, anterior intestine portion, mid intestine portion, posterior intestine portion, and rectum) (Figure 1) were fixed in Bouin’s solution (Sigma-Aldrich, St. Louis, MO, USA), dehydrated through an ethanol–xylene series, and embedded in paraffin. Sagittal sections were cut at a thickness of 4 μm, and 10 sections per region of the digestive tract were prepared for staining. Sections were stained with Hansen’s hematoxylin and 0.5% eosin (HE) for histological observation and were stained with Alcian blue (AB) at pH 2.5 and periodic acid–Schiff (PAS) for observations of mucus-secreting goblet cells [21,22]. Microscopy of the length of the mucosal folds, thickness of the muscularis externa, and characteristics of the goblet cells from different regions of the digestive tract was carried out using a light microscope (Leica, DM3000 LED, Wetzlar, Germany) attached to a camera (Dhyana 400DC, Tucsen Photonics, Fuzhou, China) with a capture program (Tucsen Mosaic version 2.2, Fuzhou, China).

### 2.3. Immunohistochemistry

Formalin-fixed tissue samples were embedded in paraffin and 4-μm sections were cut. For paraffin-embedded tissue immunohistochemistry staining, deparaffinized and rehydrated sections were incubated with 3% hydrogen peroxidase solution for 10 min at room temperature and stained with primary antibodies against cholecystokinin-8 (CCK-8) (1:20,000, Sigma-Aldrich, St. Louis, MO, USA). The stained sections were further incubated with peroxidase-conjugated secondary antibodies (EnVision+Rabbit, Dako) for 30 min, and Mayer’s Hematoxylin was applied for counterstaining. The CCK-producing cells were observed using a light microscope (Leica, DM3000 LED, Wetzlar, Germany) attached to a camera (Dhyana 400DC, Tucsen Photonics, Fuzhou, China) with a capture program (Tucsen Mosaic version 2.2, Fuzhou, China).

### 2.4. Statistical Analysis

All data were analyzed using the IBM SPSS 19 software package for Windows (SPSS Inc., Chicago, IL, USA). Data were evaluated for assumptions, including normality and homogeneity of variance, using the Shapiro–Wilk and Levene’s tests, respectively, and no violation was detected (*p* > 0.05). Statistical analyses of data were conducted using one-way analysis of variance with a 95% significance level (*p* < 0.05). When a significant treatment effect was detected, the Tukey’s honestly significant difference (HSD) test was used to assess significant differences among means.

## 3. Results

### 3.1. Histological Characteristics of the Digestive Tract

The tissue of the digestive tract can be largely divided into four layers consisting of the serosa, muscularis externa, submucosa, and muscularis mucosae from outside to inside. The internal structure characteristics of each part are described as follows. 

**Figure 2:**
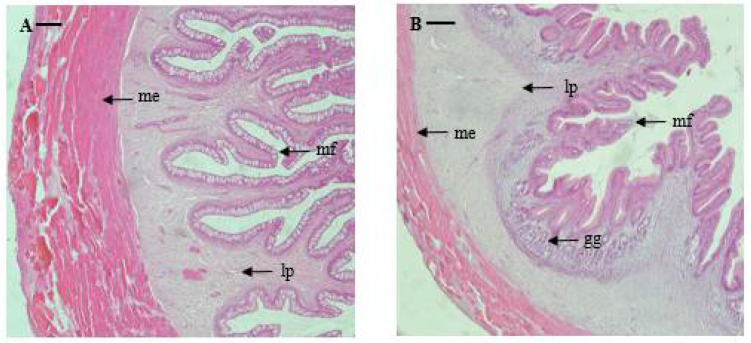

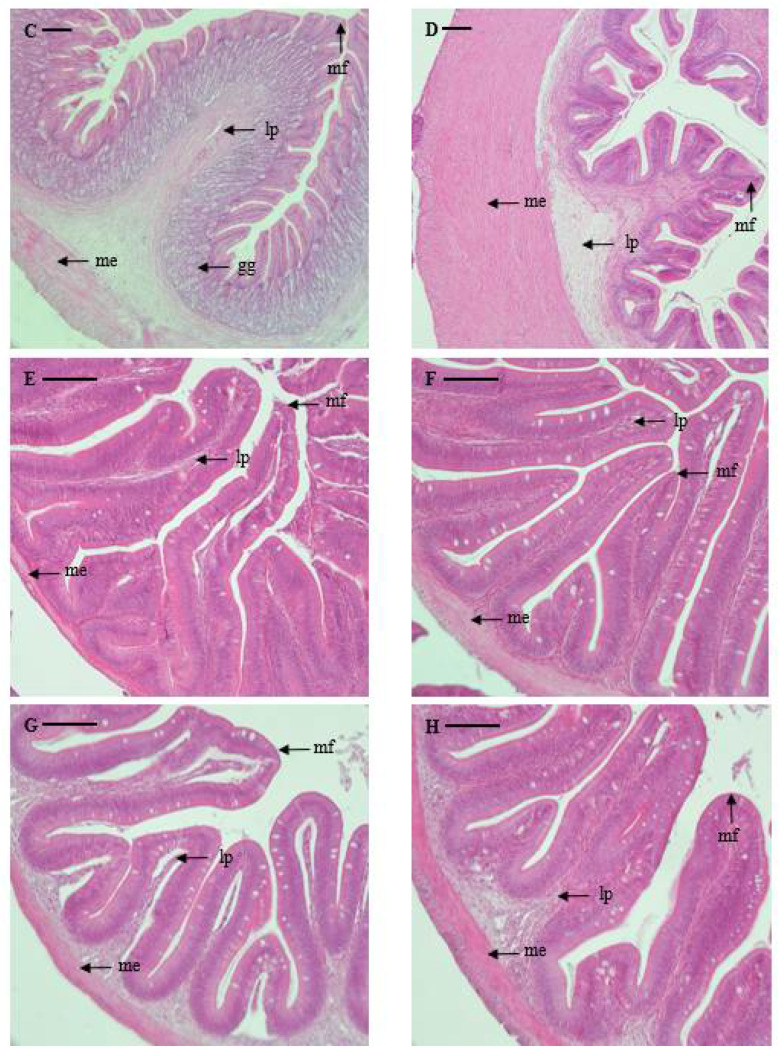

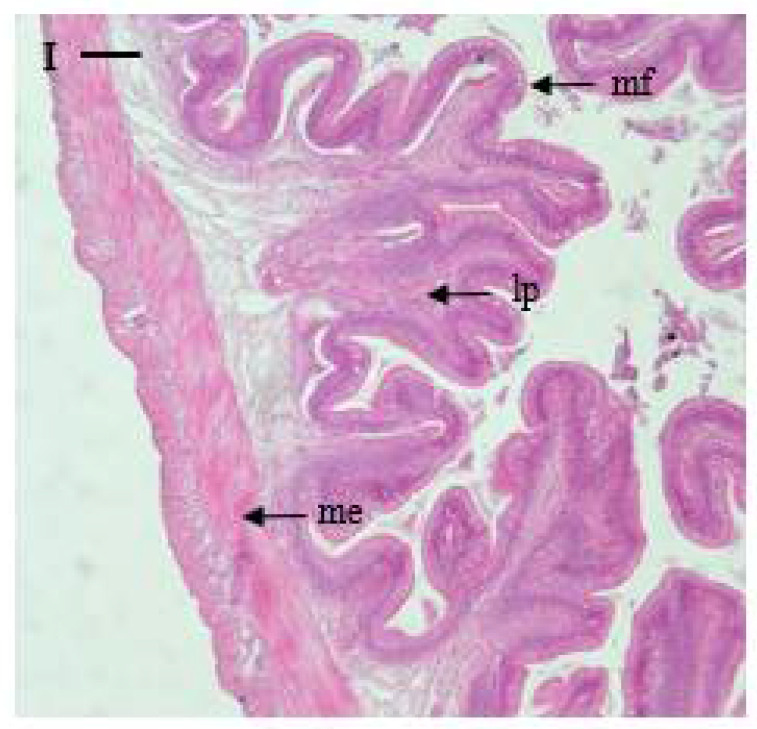
Microphotographs of cross sections of the digestive tract of the marbled flounder *Pseudopleuronectes yokohamae*: (**A**) esophagus; (**B**) cardiac stomach portion; (**C**) fundic stomach portion; (**D**) pyloric stomach portion; (**E**) pyloric caeca; (**F**) anterior intestine portion; (**G**) mid intestine portion; (**H**) posterior intestine portion; (**I**) rectum. Abbreviations: lp, lamina propria; me, muscularis externa; mf, mucosal fold; gg, gastric gland. Scale bars indicate 100 µm.

Esophagus: The serosa of the marbled flounder esophagus is thin, and the muscularis externa is widely and well-developed. The submucosa between the muscularis externa and muscularis mucosae is thin, and the lamina propria is well-developed. The mucosal folds of the esophagus are thin, long, and are arranged to form regular branches towards the lumen. The average thickness of the muscularis externa and length of the mucosal fold were 485 and 1031 μm, respectively (Table 2 and Figure 2). 

Stomach: The stomach was investigated by dividing it into cardiac, fundic, and pyloric portions, and the mucosal folds of the stomach showed a regular shape without forming branches. The serosa is thin, similar to that in the esophagus, and the muscularis externa is widely and well-developed. In the center, the gastric glands with clear lumen formation are well-developed and are distributed primarily in the fundic portion. The thickness of the muscularis externa sequentially decreases along the pyloric portion, cardiac portion, and fundic portion (603, 245, and 150 μm, respectively); while the length of mucosal folds is reversed, sequentially increasing along the fundic portion, cardiac portion, and pyloric portion (1383, 1044, and 906 μm, respectively) (Table 2 and Figure 2).

Pyloric caeca: There were six to nine pyloric caeca in marbled flounder, depending on the individual. The serosae of the pyloric caeca are so thin that it is difficult to differentiate them. The mucosal folds are irregular in shape and form branches. The degree of development of the muscularis externa is very weak compared to that of the esophagus and stomach (32 μm). The mucosal folds are shorter compared to those of the esophagus and stomach but longer than that of the intestine (669 μm) (Table 2 and Figure 2). 

Intestine: The intestine could be divided into the anterior portion, mid portion, and posterior portion according to the internal structures such as external shape, shape and length of mucosal folds, thickness of the muscularis externa, and degree of development of the epithelial layer. In the anterior portion, mucosal folds are irregular in shape and show clear formation of branches. Although the muscularis externa is very thin (57 μm), it is thicker than that of the pyloric caeca. The mucosal folds in the anterior portion are the longest (630 μm) among all intestine portions and are similar in length to those in the pyloric caeca. The mucosal folds of the mid portion are also irregular in shape, with clear formation of branches. While the muscularis externa is of a similar thickness (50.9 μm) to that of the anterior portion, the mucosal folds are shorter (486.6 μm). The mucosal folds of the posterior portion are irregular in shape and form branches. The muscularis externa is similar to those in other parts of the intestine but is thicker (70.4 μm). The mucosal folds are a similar length to those of the mid portion (458 μm) (Table 2 and Figure 2).

Rectum: The rectum is well-developed, with distinct divisions in the serosa, muscularis externa, and submucosa, and the mucosal folds are irregular and complicated in shape compared to those in the intestine, forming branches. The muscularis externa is thicker (198 μm) and the mucosal folds are longer (859 μm) compared to those in the intestine and pyloric caeca (Table 2 and Figure 2).

### 3.2. Distribution and Characteristics of Goblet Cells

**Figure 3:**
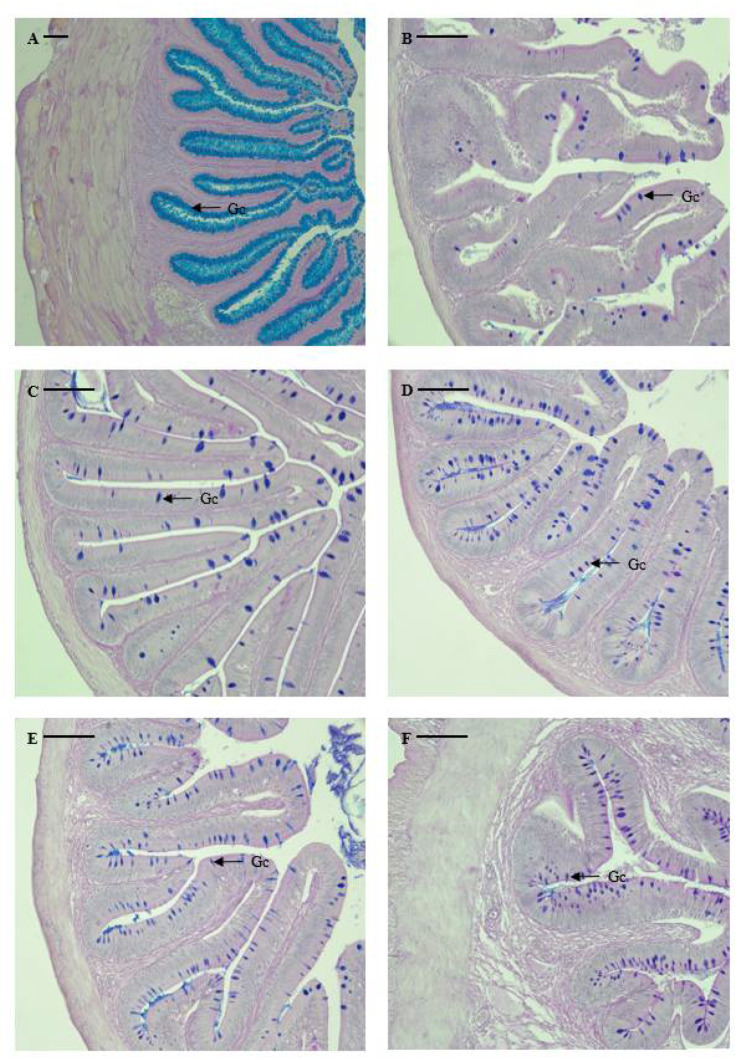
Microphotographs of mucus-secreting goblet cells in the digestive tract of marbled flounder *Pseudopleuronectes yokohamae*: (**A**) esophagus; (**B**) pyloric caeca; (**C**) anterior intestine portion; (**D**) mid intestine portion; (**E**) posterior intestine portion; (**F**) rectum. Abbreviation: Gc, goblet cell. Scale bars indicate 100 µm.

The distribution and relative frequency of goblet cells differed according to their location in the digestive tract, as shown in Table 3 and Figure 3. Notably, no goblet cells were identified in the stomach of the marbled flounder. Mucus-secreting goblet cells were identified in the esophagus (5743 cells), pyloric caeca (300 cells), anterior intestine portion (650 cells), mid intestine portion (528 cells), posterior intestine portion (467 cells), and rectum (1943 cells). Among the digestive tract regions, goblet cells had significantly higher distribution in the esophagus, followed by the anus (*p* < 0.05). Goblet cells were more highly distributed in the intestine than in the pyloric caeca, and the number of goblet cells decreased when moving from the anterior to the posterior intestine portion. However, there was no significant difference in the number of goblet cells between the intestine and pyloric caeca (*p* > 0.05). Esophageal goblet cells were densely distributed and filled the lumen, portraying a spherical or oval shape. Goblet cells from the anterior intestine portion to the anus were distributed throughout the mucosal folds in a spherical or oval shape. 

### 3.3. Distribution and Characteristics of CCK-Producing Cells

**Figure 4:**
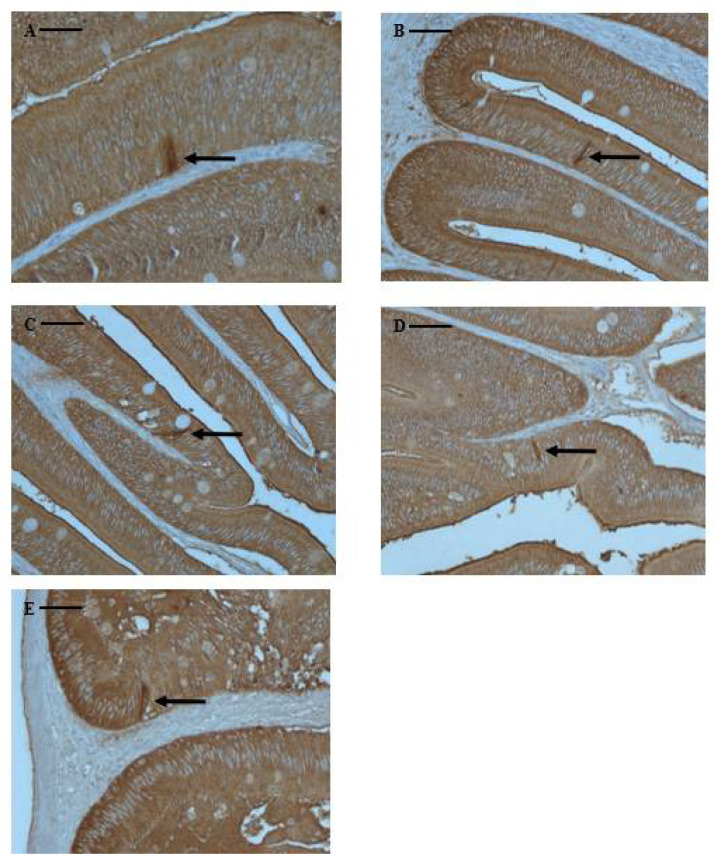
Microphotographs of CCK-producing cells in the digestive tract of marbled flounder *Pseudopleuronectes yokohamae*. (**A**) pyloric caeca; (**B**) anterior intestine portion; (**C**) mid intestine portion; (**D**) posterior intestine portion; (**E**) rectum. Arrows indicate CCK-producing cells. Scale bars indicate 40 µm.

CCK-producing cells are typical endocrine-like cells, characterized by a long spindle shape with a narrow apex pointing towards the intestinal lumen. Although marbled flounder CCK-producing cells were not found in the esophagus or stomach, they were found at various frequencies in the pyloric caeca and intestine, extending to the rectum. CCK-producing cells were identified in the pyloric caeca (65 cells), anterior intestine portion (62 cells), mid intestine portion (13 cells), posterior intestine portion (8 cells), and rectum (4 cells). They were primarily distributed in the pyloric caeca and anterior intestine portion, showing a lower frequency towards the rectum (Table 3 and Figure 4).

## 4. Discussion

To clarify the physical and chemical characteristics of the marbled flounder digestive tract, this study conducted morphological, histological, and histochemical analyses. The length of the intestine in the digestive tract of teleosts is an important parameter to distinguish the dietary patterns of fish. Al-Hussaini (1947) [23] divided fish by their feeding habit based on their RLG values: carnivorous fish, 0.5~2.4; omnivorous fish, 1.3~4.3; and herbivorous fish, 3.7~6.0. In addition, Dasgupta (2004) [24] observed that the mean RLG values were 0.7 in carnivorous fish, 1.4 in omnivorous fish, 4.8 in herbivorous fish, and 3.7 in plankton feeder fish. The RLG value of marbled flounder in this study was found to be 1.54 ± 0.10, which was similar to that of omnivorous fish; however, the shape and anatomical structure of the digestive tract showed similar characteristics to those of carnivorous fish. According to the results of a recent study, the feeding habits of marbled flounder are in fact close to those of carnivorous fish, as their main prey is Polychaeta (45.3%) and Gastropoda (23.2%) [25].

The esophagus of the marbled flounder showed a branched shape with mucosal folds along the longitudinal axis, similar to the esophagus of many teleosts [26], and a stratified epithelial layer consisting mainly of epithelial and secretory cells [27,28]. This structure suggests that the esophagus of the marbled flounder can be expanded for the digestion and transportation of food. In the esophagus, the number of goblet cells was significantly higher than in other digestive tract tissues. Goblet cells secrete mucus to protect the mucosal epithelial layer and facilitate the transport of food substances and excretion into the digestive tract [29]. Therefore, it is suggested that the presence of many goblet cells in the esophagus of the marbled flounder is for lubricating and protecting the esophagus.

Digestion in the stomachs of teleosts can be divided into physical and chemical digestion. Physical digestion varies depending on the development of the muscularis externa and muscularis mucosa, and chemical digestion varies depending on the degree of gastric gland development [30,31]. Fish with poorly developed muscularis mucosa but well-developed muscularis externa are suited for digesting a large amount of soft food. In contrast, fish with poorly developed muscularis externa but well-developed muscularis mucosa are suited for ingesting a small amount of hard food [32]. In the case of the marbled flounder, the muscularis externa of the cardiac and fundic portions of the stomach has weak muscularis externa development compared to that of the pyloric portion, but the muscularis mucosa of the cardiac and fundic portions has well-developed gastric glands for chemical digestion. In addition, although there are no gastric glands in the pyloric portion, the muscularis externa is very well-developed, suggesting that the marbled flounder has a stomach with a structure suitable for eating a small amount of hard food. 

The pyloric caeca of fish are an evolutionary strategy to increase the total surface area of the digestive epithelium, without increasing the length or thickness of the intestine [33]. Pyloric caeca are found in approximately 60% of teleosts, especially in carnivorous species [34]. The number of pyloric caeca in the marbled flounder was found to be six to nine, and the morphological structure of the digestive tract was similar to those of carnivorous fish. However, since little research has been conducted on the correlation between the number of pyloric caeca and size of the teleost intestine and the relationship between the number of pyloric caeca and digestive physiology, additional research is needed. 

The shape of the mucosal folds of the intestine is more complex than that of the esophagus or stomach, and these structural characteristics increase the dwell time of food in the intestine and increase its surface area, improving digestion function and absorption of nutrients [35]. In other words, the mucosal folds of the fish intestine are well developed (more complex and more branched) and have higher digestive activity [31]. Mucosal folds in the anterior, mid, and posterior intestine portions of the marbled flounder showed similar branched patterns; however, the mucosal folds became shorter moving from the anterior intestine portion to the posterior intestine portion, suggesting that more digestion takes place in the anterior intestine portion than in the posterior intestine portion. 

Previous studies investigating the histochemical characteristics of mucus-secreting cells found that the shape, size, and distribution of goblet cells in the digestive tract differ depending on the fish species and digestive tract structure [35,36,37,38]. The goblet cells in the digestive tract of marbled flounder showed a different shape and distribution compared to those of other fish. For marbled flounder, goblet cells were irregularly distributed without a regular arrangement from the upper part to the lower part of the mucosal folds, and the shape of goblet cells appeared to vary from spindle to oval. The number of goblet cells in the intestine of the marbled flounder decreased from the anterior to the posterior portion. Lee and Chin (1995) [32] reported that the distribution of goblet cells in the gut of stomachless fish increased towards the posterior intestine portion, and Hur et al. (2013) [39] reported that the distribution of goblet cells decreased towards the posterior intestine portion in stomach fish. Judging from the results of previous studies, marbled flounder displayed a digestive characteristic typical of stomach fish.

The final absorption of water, ions, and proteins takes place in the teleost rectum [9] and many mucin-containing epithelial cells exist in the rectum to contribute to the passage of feces, protection of the epithelium, and final absorption of substances [35]. The histological and histochemical analysis results revealed that the marbled flounder rectum was composed of a complex columnar shape, similar to that of other teleost fish. In the epithelial lining, epithelial cells coated in secretions of neutral and acidic mucus and a number of goblet cells were observed, which were judged to contribute to the absorption of substances generally performed in the rectum. 

In fish, CCK cells are present among the endocrine cells of digestive tract and within the central and peripheral nervous system [40,41]. These cells influence digestive processes by regulating food intake through peripheral satiety signals [42]. Marbled flounder CCK cells were distributed throughout the digestive tract, including the pyloric caeca, intestine, and rectum, and CCK cell frequency was highest in the pyloric caeca and anterior portion of the intestine. CCK cells are found in various fish species, including flat fish species [43,44], and distribution patterns similar to that of the marbled flounder CCK cells were observed in the digestive tracts of hairychin goby *Sagamia geneionema* [30], blacktip grouper *Epinephelus fasciatus* [39], silver catfish *Rhamdia quelen* [45], dorado *Salminus brasiliensis* [46], and Neotropical freshwater fish *Prochilodus lineatus* [47]. Considering the biological roles of CCK cells, their distribution pattern in marbled flounder suggests that the pyloric caeca and anterior intestine portion of the intestine play an important role in gallbladder contraction, pancreatic enzyme secretion, stimulation of gastrointestinal motility, and inhibition of gastric emptying functions [48]. However, the distribution characteristics of neuroendocrine cells, including those that secrete CCK, in fish digestive tracts do not show common distribution characteristics among fish species and dietary patterns [29,49,50].

## 5. Conclusions

The anatomical and functional structures of the marbled flounder digestive tract were investigated and considered to closely represent the morphology of the carnivorous fish digestive tract. Through investigating the physical and chemical characteristics of the digestive tract, basic information regarding the physical characteristics and nutritional studies of food suitable for the marbled flounder were provided. However, studies on the characteristics of goblet cells according to dyeing properties, absorptive cells, and endocrine cells distributed in the digestive tract should be conducted in more detail at the ultrastructural level.

## Figures and Tables

**Table 1:** Body length, body weight, digestive tract length, and relative length of gut of the marbled flounder *Pseudopleuronectes yokohamae*.

	Marbled Flounder
Sample number	20
Body length (cm)	14.6 ± 0.5
Body weight (g)	56.6 ± 5.1
Digestive tract length (cm)	22.5 ± 2.0
Relative length of the gut	1.54 ± 0.10

Values are the means ± standard deviation (*n* = 20).

**Table 2:** Histological features of the digestive tract of the marbled flounder *Pseudopleuronectes yokohamae*.

	Muscularis Externa Thickness (μm)	Mucosal Fold Length (μm)
Esophagus	483.5 ± 23.1	1031.7 ± 26.0
Cardiac stomach portion	245.3 ± 31.8	1044.3 ± 90.4
Fundic stomach portion	150.1 ± 3.0	1383.3 ± 51.3
Pyloric stomach portion	603.5 ± 40.3	906.8 ± 45.0
Pyloric caeca	32.2 ± 1.2	669.4 ± 34.0
Anterior intestine portion	57.4 ± 8.6	630.7 ± 21.9
Mid intestine portion	50.9 ± 6.2	486.5 ± 20.2
Posterior intestine portion	70.4 ± 4.1	458.6 ± 34.1
Rectum	198.0 ± 10.8	859.5 ± 49.6

Values are the mean ± standard error (*n* = 20)

**Table 3:** Numbers of goblet cells and CCK-producing cells in different digestive tract regions of the marbled flounder *Pseudopleuronectes yokohamae*.

	Numbers/Tissue Section	
Tissue Section	Goblet Cells	CCK-Producing Cells
Esophagus	5743 ± 107 ^a^	None
Cardiac stomach portion	None	None
Fundic stomach portion	None	None
Pyloric stomach portion	None	None
Pyloric caeca	300 ± 32 ^c^	65 ± 6 ^a^
Anterior intestine portion	650 ± 86 ^c^	62 ± 3 ^a^
Mid intestine portion	528 ± 59 ^c^	13 ± 1 ^b^
Posterior intestine portion	467 ± 72 ^c^	8 ± 2 ^b^
Rectum	1943 ± 144 ^b^	4 ± 1 ^b^

Values are the mean ± standard error (*n* = 20). Values followed by differing superscript letters are significantly different (Tukey’s HSD test, *p* < 0.05).

## Data Availability

Not applicable.
